# Genotypic peculiarities of a human brucellosis case caused by *Brucella suis* biovar 5

**DOI:** 10.1038/s41598-023-43570-4

**Published:** 2023-10-03

**Authors:** Hanka Brangsch, Matthias A. Horstkotte, Falk Melzer

**Affiliations:** 1 Friedrich-Loeffler-Institut – Federal Research Institute for Animal Health, Institute of Bacterial Infections and Zoonoses, Jena, Germany; 2 Labor Lademannbogen MVZ GmbH, Hamburg, Germany

**Keywords:** Bacterial infection, Infectious-disease epidemiology, Infectious-disease diagnostics

## Abstract

Human brucellosis cases are rare in non-endemic countries, such as Germany, where infections are predominantly caused by *Brucella melitensis*. The German National Reference Laboratory for Bovine, Porcine, Ovine and Caprine Brucellosis received a suspected *Brucella* sp. isolate from a patient for identification. Bacteriological tests and PCR-based diagnostics showed the isolate to be *B. suis*, but did not yield cohesive results regarding the biovar. Whole genome sequencing and subsequent genotyping was employed for a detailed characterization of the isolate and elucidating the reason for failure of the diagnostic PCR to correctly identify the biovar. The isolate was found to be *B. suis* bv. 5, a rare biovar with limited geographical distribution primarily found in the Northern Caucasus. Due to a deletion in one of the target regions of the diagnostic PCR, the isolate could not be correctly typed. Based on in silico genotyping it could be excluded that the isolate was identical to one of the *B. suis* bv. 5 reference strains. Here, we report a rare case of a *B. suis* bv. 5 field isolate. Furthermore, by reporting this finding, we want to make practitioners aware of possible misinterpretation of PCR results, as it cannot be excluded that the detected deletion is common among the *B. suis* bv. 5 community, as there is currently a lack of field isolates.

## Introduction

Brucellosis is a zoonotic disease that has had many names over the centuries, such as Malta fever or Mediterranean fever, before its causative agent was discovered in 1887 by the British microbiologist David Bruce, after whom it was later named *Brucella*, and the disease was called “brucellosis”^1,2^. The primary hosts for the pathogen are animals, and there is a preference of the various *Brucella* spp. for specific animal species. However, close contact with infected animals can lead to infection of non-primary host species, like humans. Especially in endemic regions, like the Mediterranean Basin^3^, the risk of contracting brucellosis can be quite high. Contact with animal abortion material, handling of carcasses, consumption of contaminated, raw animal products such as unpasteurized milk, as well as laboratory work are the main sources of infection. Thus, veterinarians, hunters, cooks and laboratory workers are some of the occupational groups with an increased risk of a *Brucella* infection^4–6^. The identification of the disease as brucellosis is often difficult due to unspecific symptoms, e.g. usually fever, sweat, headache, arthralgia and weight loss, which may occur episodically for months or even years after the infection^5–9^. Therapeutically, diagnosis is followed by a long antibiotic treatment and relapses are frequent, especially in endemic, rural areas where medical care often cannot be sufficiently ensured^7,10,11^.

The global number of human infections per year can hardly be estimated reliably, as brucellosis is often underreported^12^. In the literature, 500,000 cases per year is the most cited number, however, it was pointed out, that this number is not reliable and a product of incorrect citation throughout brucellosis literature^1^. Based on statistical modelling using international healthcare data, a conservative estimate gives an annual incidence of 2.1 million^13^. Since 2017, a decline of confirmed human brucellosis cases is observed in the European Union (EU), from 0.09 confirmed cases per 100,000 population in 2017 to 0.03 in 2021^14^, e.g. in 2019, 310 human brucellosis infections have been reported in the EU, which accounts for a notification rate of 0.06 cases per 100,000 population^15^.

With regard to human brucellosis, *B. melitensis*, *B. abortus* and *B. suis* are the most commonly identified species. *B. melitensis* has the highest potential for causing brucellosis in humans and is the most frequently isolated species from brucellosis patients in the EU^15–17^. On the contrary, reports on human infections with *B. suis* in the EU are rare. In the period of 2006 to 2018, the vast majority (91%) of notified human brucellosis cases in Germany were caused by *B. melitensis*, whereas only a single infection with *B. suis* was reported^5^. In the latter case, *B. suis* bv. 1 was identified as the causative agent and the infection resulted most likely from consumption of contaminated meat, possibly from Argentina^7^.

The species *B. suis* is divided into five biovars based on phenotypic properties. They also exhibit different host preferences and regional prevalences. The biovars 1, 2 and 3 are commonly found in wild boars and domestic pigs, with the former two biovars also infecting hares^4,18–21^. Biovar 2 is the most frequently reported biovar of *B. suis* in Europe. Infections with *B. suis* bv. 4 have occurred mainly in northern America affecting reindeers and caribous^22^. In contrast, *B. suis* bv. 5 has exclusively been reported from the Northern Caucasus and South West Siberia, where it primarily infects rodents^23,24^. In Germany, *B. suis* bv. 2 is the only biovar that circulates among animals, as it is frequently reported in wild boars and domestic pigs, also from neighbouring countries^25–28^.

Although different serological tests for brucellosis diagnosis are available, the isolation of the bacterium from the patient gives the only conclusive evidence. For the quick identification of *Brucella* species directly from *Brucella* suspected specimen, PCR assays have been developed. The most widely used approaches are AMOS and Bruce-ladder PCR. With the former, *Brucella abortus* biovars 1, 2, and 4, *Brucella melitensis*, *Brucella ovis*, and *Brucella suis* bv. 1 can be detected. The PCR is based on species-specific chromosomal locations of IS711, whose position differs among *Brucella* species^29^ and it was later enhanced for the additional differentiation of vaccine strains *B. abortus* S19 and RB15^30^. However, with this approach, not all *Brucella* species can be detected. Thus, a different assay was developed based on marker genes, with which all classical *Brucella* sp., *B. neotomae*, *B. pinnipedialis*, *B.* *ceti* and some vaccine strains could be identified, called Bruce-ladder PCR^31,32^. For further PCR-based discrimination of *B. suis* biovars and the closely related *B. canis*, a Suis-ladder PCR was developed^33^. In this multiplex PCR using four primer pairs, the *B. suis* biovars can be identified based on varying product lengths in some of the investigated loci.

For epidemiological studies aiming at deciphering the relationship between strains, genome sequencing and bioinformatic analysis should be employed. Already before the advent of sequencing techniques, the high genomic similarity of *Brucella* sp. has been described^34^. Thus, the common thresholds for species delineation in the analysis of the average nucleotide identity (ANI) analysis, does not apply^35^. For example, the ANI of *B. suis* strains is > 99.85%, i.e. individual strains may differ in as little as 0.15% of their genome from each other. Therefore, single nucleotide substitutions have to be employed for the reconstruction of strain similarities, that help identifying the infection source^36,37^. This analysis heavily depends on the availability of genome sequence data, with which an isolate’s sequence can be compared. In particular for *B. melitensis*, single nucleotide polymorphism (SNP) typing helped identifying the potential geographic origin of the infection of travel-associated human brucellosis cases in Germany^38^.

Here, we report on the genotypic and phenotypic peculiarities of an isolate from a rare case of human brucellosis, for which *B. suis* bv. 5 was identified as causing agent. The unambiguous identification of the pathogen was hampered by the fact that bacteriological diagnostics based on standard differential tests indicated that this isolate was a strain of *B. suis* bv. 5, but one band in the Suis-ladder PCR did not yield the expected size for this biovar. Thus, genome sequencing-based analysis, which is now routinely used for diagnostic purposes, had to be used for identification and genotyping of the human isolate, revealing its affiliation to biovar 5.

## Methods

### Isolate origin and case description

In 2008, a suspected *Brucella* sp. isolate was sent to the National Reference Laboratory (NRL) for Bovine, Porcine, Ovine and Caprine Brucellosis at Friedrich-Loeffler-Institut for confirmation and further typing. The following information on the sample was provided by the sender: The isolate was obtained in Germany from a blood culture of a 71-year old male patient with a septic clinical picture with fever episodes up to 41 °C and nearly fever-free intervals. The isolate was given the FLI number 08RB3647.

### Phenotypic methods

The isolate was cultivated on nutrient agar (Merck, Germany) and bacteriological tests for *Brucella* differentiation were conducted as described elsewhere^39^, i.e. testing for catalase, oxidase and urease activity, CO_2_ requirement, H_2_S production, agglutination assays, phage lysis and tolerance to the dyes fuchsin and thionin. Further, VITEK GN ID card (bioMérieux, Marcy-l′Etoile, France) was used for genus confirmation.

### DNA extraction and PCR

DNA was isolated for molecular investigations using the High Pure PCR Template Preparation Kit (Roche Molecular Systems, Pleasanton, CA, USA). For identifying the species, AMOS and Bruce-ladder PCR were conducted as described before^29,32,40^. Suis-ladder PCR, hereafter referred to as *B. suis* ladder PCR, according to López-Goni et al.^33^, was used for strain differentiation on the biovar level. For the latter, DNA from reference strains *B. suis* bv. 1 strain 1330, *B. suis* bv. 2 strain Thomsen, *B. suis* bv. 3 strain 686, *B. suis* bv. 4 strain 40 and *B. suis* bv. 5 strain 513 were used as references. Cultivation and DNA extraction was conducted as described for strain 08RB3647.

### Genome sequencing and assembly

For Illumina short-read sequencing, a DNA library was prepared using the Nextera XT library preparation kit (Illumina Inc., San Diego, CA, USA), and sequenced on a MiSeq device in paired-end mode using v3 chemistry. Additionally, long-read sequencing was performed by Eurofins Genomics Germany GmbH (Ebersberg, Germany) on a PacBio RSII instrument. The raw data from both technologies were used for de novo genome assembly in a hybrid approach using Unicycler v.0.4.8^41^. Starting positions of the chromosomes were adjusted by circlator v1.5.5^42^ and the assembly aligned to *B. suis* bv. 5 CVI_73 (GCF_014884485.1) with Mauve ^43^ for checking for assembly errors. QUAST v5.0.2^44^ was used for further assessing assembly statistics and genome annotation was performed by Prokka v.1.14.5^45^. Raw sequencing data and the assembled genome were deposited under the BioProject number PRJEB62596.

### In silico* genome characterization*

Using the in_silico_pcr script by Egon A. Ozer (v0.5.1) (https://github.com/egonozer/in_silico_pcr) with allowing one mismatch in the primer binding site (option “-m”) and primers by López-Goni et al.^33^, an in silico* B. suis* ladder PCR was conducted for confirmation of the in vitro result. The products were aligned using MAFFT v7^46^ and visualized by pyBoxShade (https://github.com/mdbaron42/pyBoxshade). The average nucleotide identity of the de novo genome to the genomes of the strains *B. suis* bv. 1 1330 (GCF_000223195.1), *B. suis* bv. 2 Thomsen (GCA_000018905.1), *B. suis* bv. 3 686 (GCF_000740255.1), *B. suis* bv. 4 40 (GCF_000160275.1), *B. suis* bv. 5 513 (GCF_000157755.1) and *B. suis* bv. 5 CVI_73 (GCF_014884485.1) was determined by fastANI v1.1^47^.

The genotype of the investigated strain was determined by in silico multi-locus sequence typing (MLST) and multiple locus variable number of tandem repeats analysis (MLVA) as allele-based methods. The tools mlst v2.19.0 (https://github.com/tseemann/mlst) with the MLST-9 scheme by from PubMLST^48^, and MISTReSS (https://github.com/Papos92/MISTReSS) employing the MLVA-16 scheme by Le Flèche et al.^49^ and Al Dahouk et al.^50^ were used for these purposes, respectively. The MLVA profile was used as query in a search for identical or similar profiles in the MLVAbank database (https://microbesgenotyping.i2bc.paris-saclay.fr/)^51^ (accessed on 5th December 2022). In addition, single nucleotide polymorphism (SNP) analysis was carried out using Snippy v.4.6.0 (https://github.com/tseemann/snippy), with *B. suis* bv. 5 CVI_73 as reference genome. Data of *B. suis* reference strains and other closely related species (Supplementary Table S1) from the NCBI Sequence Read Archive were also included in this analysis. The resulting alignment of core genome SNPs was used as input for maximum likelihood analysis by RAxML v8.2.12^52^ employing the GTRGAMMA model of rate heterogeneity and optimization of substitution rates and the final tree was visualized by FigTree v1.4.3 (http://tree.bio.ed.ac.uk/soft-ware/figtree/).

The novel genome was screened for potential antimicrobial resistance genes by comparing the sequences to entries in three databases (Resfinder^53^, CARD ^54^, AMRFinder^55^) by ABRicate v0.8.10 (https://github.com/tseemann/abricate). Additionally, the VFanalyzer pipeline of the VFDB^56^ was employed for detecting genes related to virulence (accessed on 1st December 2022).

### Antimicrobial susceptibility testing

Analysis of antimicrobial resistance was conducted in user-defined commercial microdilution plates (Sensititre; Thermo Scientific, Waltham, MA, USA) containing antibiotics in the following final concentration ranges: chloramphenicol (0.5–16 µg/ml), ciprofloxacin (0.002–4 µg/ml), doxycycline (0.004–8 µg/ml), gentamicin (0.004–8 µg/ml), levofloxacin (0.004–4 µg/ml), rifampicin (0.125–8 µg/ml), streptomycin (0.008–16 µg/ml), tetracycline (0.004–8 µg/ml), trimethoprim/sulfamethoxazole (0.002/0.04–4/76 µg/ml) and ceftriaxone (0.004–0.12 µg/ml). For inoculation, the strain was cultivated on Columbia blood agar (Thermo Scientific, Waltham, MA, USA) for 48 h at 37 °C under aerobic conditions. Subsequently, a 1:10 diluted 0.5 McFarland suspension was prepared in 0.9% NaCl solution, of which 200 µl were added to 11 ml cation-adjusted Mueller Hinton broth (CAMHB) (BD Diagnostics, Franklin Lakes, NJ, USA). To each well of the microdilution plate, 100 µl were added and the plate was closed and incubated at 37 °C for 48 h under aerobic conditions. The minimum inhibitory concentration (MIC) endpoints were evaluated visually using an inverted mirror. For susceptibility evaluation the epidemiological cut-off values (ECOFFs) of the European Committee on Antimicrobial Susceptibility Testing (EUCAST) for *B. melitensis* were used (https://mic.eucast.org/; accessed on 06.09.2023), as no official values exist for *B. suis*.

## Results

### Differential testing and PCR

Based on differential testing at the NRL, strain 08RB3647 can be described as Gram-negative, non-motile coccoid rods. The strain did not require CO_2_ and was negative for haemolysis and H_2_S production but positive for the production of catalase, oxidase and urease (Table 1). Biochemical identification by VITEK GN ID card indicated *Brucella melitensis*. In the serum agglutination the strain showed agglutination with anti-M monospecific serum only, which is characteristic for *B. suis* bv. 5. Further, tests for lysis by phages gave overall negative results for all of the tested phages, although small, single plaques could be observed within the tested zones. Only at an increased concentration lysis was observed for phage Tbilisi (Tb).

**Table 1:** Results of differential tests of field isolate 08RB3647.

Strain	CO_2_ requirement	H_2_S production	Oxidase	Catalase	Urease activity	Growth on		Agglutination with monospecific sera				Lysis by phages		
						Thionin	Fuchsin	A	M	R	F25	Wb	Tb	10^4^ Tb
08RB3641	−	−	+	+	+	+	−	−	+	−	− *	− *	− *	+

*No overall lysis, but tiny plaques.

**Figure 1:**
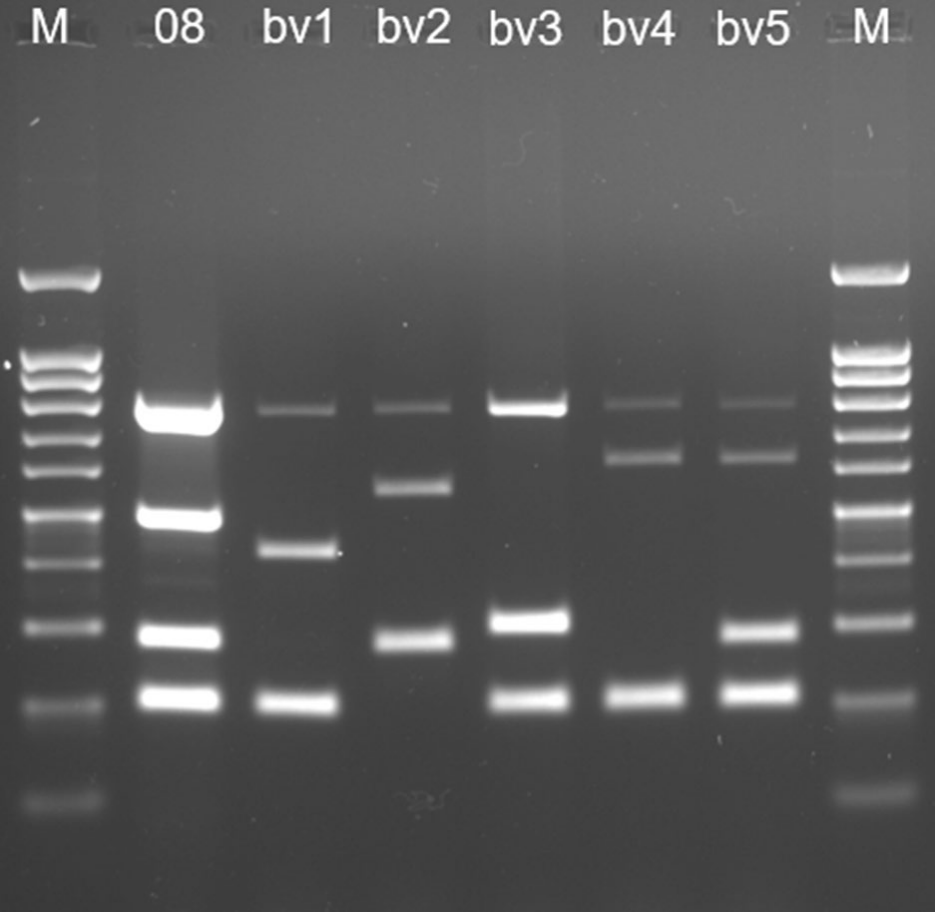
Agarose gel of *B. suis* ladder PCR products of strain 08RB3647 (08) and *B. suis* reference strains (bv1—*B. suis* bv. 1 1330; bv2—*B. suis* bv. 2 Thomsen; bv3—*B. suis* bv. 3 686; bv4—*B. suis* bv. 4 40; bv5—*B. suis* bv. 5 513). M indicates the 100 bp ladder.

The Bruce-ladder PCR confirmed that the isolate 08RB3647 was indeed a *B. suis* strain, as it gave the expected band pattern that corresponds to this species. Subsequently, the *B. suis* biovar should also be determined by PCR. Strain 08RB3641 was negative in the AMOS PCR, which ruled out its identity as *B. suis* bv. 1. However, the result of the *B. suis* ladder PCR was unexpected, as a band of appr. 500 bp length could be observed (Fig. 1), which was not characteristic for any of the known *B. suis* biovars.

### Genome sequencing and characterisation

Sequencing yielded short reads of 247 bp for 287-fold coverage in case of Illumina technology and 80,359 reads with a mean length of 9678 bp giving an average coverage of 211-fold for PacBio technology. By combining reads from both techniques in a de novo hybrid assembly approach, two chromosomes with sizes of 2,131,613 bp and 1,187,908 bp could be assembled (Table 2). The average genomic GC content was 57.25% and 3091 coding regions were predicted. These values were in accordance with those of the genome assembly of *B. suis* bv. 5 CVI_73, which was the only complete *B. suis* bv. 5 genome available at NCBI at the time of writing.

**Table 2:** Characteristics of the genome assemblies of strain 08RB3647 and *B. suis* bv. 5 CVI_73 (GCF_014884485.1).

	08RB3647	*B. suis* bv. 5 CVI_73
Contigs	2	2
Assembly size	3,319,521 bp	3,319,604 bp
Chromosome I	2,131,613 bp	2,131,677 bp
Chromosome II	1,187,908 bp	1,187,927 bp
GC content	57.25%	57.25%
CDS	3091	3131
rRNA	9	9
tRNA	55	54
tmRNA	1	1

To confirm species identification and to clarify the identification on biovar level, the average nucleotide identity (ANI) of the de novo assembly to *B. suis* reference strains and the field isolate CVI_73 was determined (Table 3). Strain 08RB3647 exhibited high ANI values to all *B. suis* biovars (ANI > 99.7%), but the highest identity was observed to *B. suis* bv. 5 strains, particularly strain CVI_73, which differed by only 0.009%, confirming the affiliation of strain 08RB3647 to biovar 5.

**Table 3:** Average nucleotide identity (ANI) between strain 08RB3647 and *B. suis* reference strains.

Accession	Species	Biovar	Strain	ANI with 08RB3647 (%)
GCF_000223195.1	*B. suis*	1	1330	99.794
GCA_000018905.1	*B. suis*	2	Thomsen	99.754
GCF_000740255.1	*B. suis*	3	686	99.771
GCF_000160275.1	*B. suis*	4	40	99.750
GCF_000157755.1	*B. suis*	5	513	99.934
GCF_014884485.1	*B. suis*	5	CVI_73	99.991

**Figure 2:**
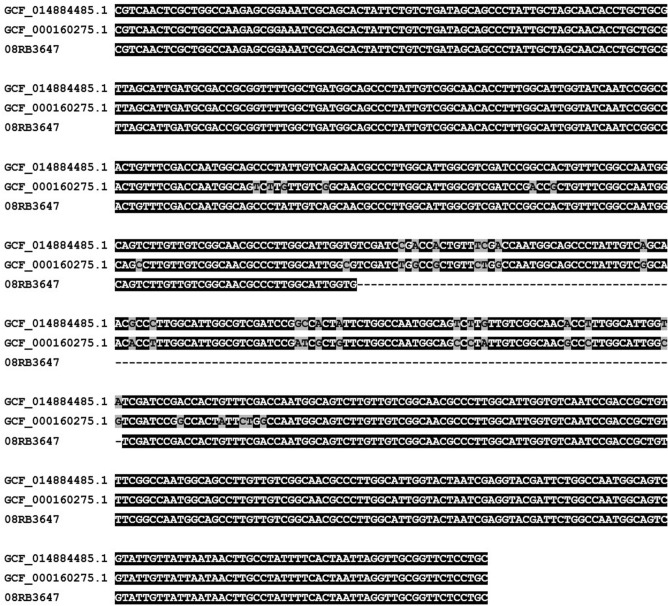
Alignment of in silico PCR products of BMEI0205-like locus in *B. suis* bv. 5 CVI_73 (GCF_014884485.1), *B. suis* bv. 4 40 (GCF_000160275.1) and strain 08RB3647. Black shading indicates identity of bases, while grey shading indicates differences between the sequences.

The high genomic concordance to *B. suis* bv. 5 strains raised the question why the *B. suis* ladder PCR did not yield the expected band of 614 bp length, but a shorter band. Therefore, this PCR was repeated in silico. The strains *B. suis* bv. 5 513 and CVI_73 yielded the expected patterns with products of 774 bp, 614 bp, 278 bp and 197 bp in length, whereas in strain 08RB3647 the product of primers BMEI0205f/r was truncated exhibiting a size of 488 bp, due to a 126 bp deletion in a locus homologous to BMEI0205 (Fig. 2). Apart from this deletion, the products of strain CVI_73 and strain 08RB3647 were identical for this locus, whereas nucleotide substitutions were observed in the product of *B. suis* bv. 4 strain 40, which yielded a band of identical size.

Regarding potential antimicrobial resistance and virulence genes, no particularities could be observed. Genes encoding a multidrug efflux RND transporter (*bepD-G*) and the integral membrane protein MprF were identified in strain 08RB3647 as well as in the other *B. suis* bv. 5 strains. Likewise, the results for potential virulence-associated genes was identical in the tested strains.

### Genotyping

In silico typing showed that strain 08RB3647 belonged to MLST-9 sequence type 19, like *B. suis* bv. 5 strain 513. Regarding the MLVA-16 profile, both strains differed in eight loci (Table 4), whereas *B. suis* bv. 5 strain CVI_73 was almost identical to the reference strain 513, differing only in one locus. When searching the MLVAbank for strains with a similar profile to strain 08RB3647, no strains with identical MLVA profiles were found. The most similar strains were REF 513 and BCCN#R29, both of which belong to *B. suis* bv. 5, probably derivatives of the reference strain 513, and differ from strain 08RB3647 in eight and six loci, respectively.

**Table 4:** MLVA-16 profiles determined either in silico (08RB3647, strain 513, CVI_73) or taken from MLVAbank (BCCN#R29; REF 513).

Strain	Bruce06	Bruce08	Bruce11	Bruce12	Bruce42	Bruce43	Bruce45	Bruce55	Bruce18	Bruce19	Bruce21	Bruce04	Bruce07	Bruce09	Bruce16	Bruce30
08RB3647	1	2	7	17	1	2	5	6	8	38	9	7	7	9	9	7
CVI_73	1	2	9	17	1	2	5	5	7	43	9	9	5	3	9	5
Strain 513	1	2	9	17	1	2	5	5	7	43	9	9	5	3	11.5	5
BCCN#R29	1	2	9	14	1	2	5	6	8	43	9	7	5	9	5	6
REF 513	1	2	7	14	1	2	5	5	7	43	9	9	5	3	9	5

**Figure 3:**
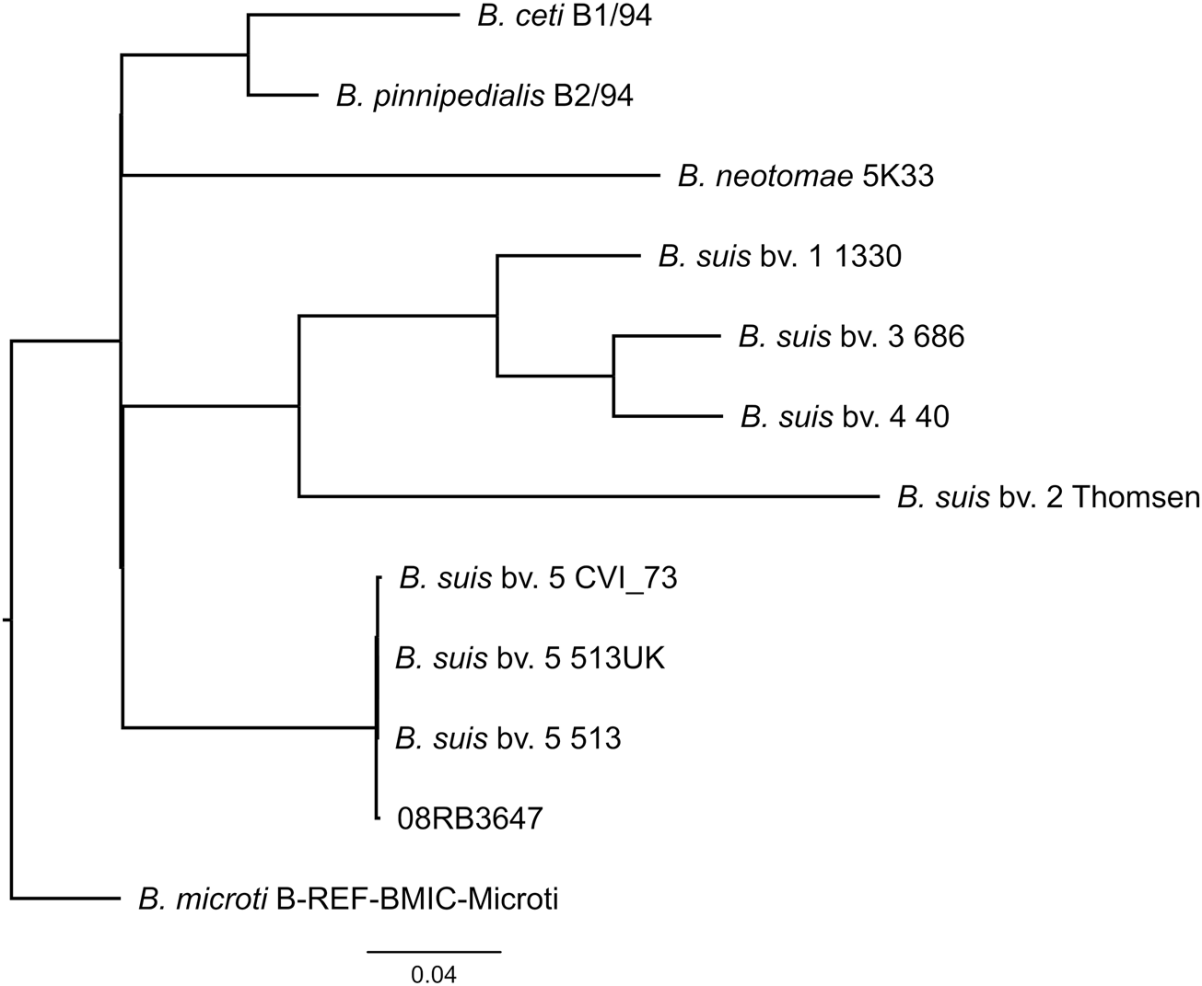
Maximum-likelihood tree based on cgSNP alignment including members of the core clade of the *Brucella* phylogeny. The scale bar indicates the number of nucleotide substitutions per site.

For a more detailed genomic comparison, core genome SNP analysis was conducted, including reference strains of *B. suis* and other *Brucella* spp. (Fig. 3). The human isolate 08RB3647 clustered with other *B. suis* bv. 5 strains, which originate from animals. However, 35 and 63 SNPs differed between strain 08RB3647 and the reference strains 513/ 513UK and the field strain CVI_73 from Slovakia, respectively. The latter differed in 28 SNPs from the references, which were both identical in cgSNP analysis. Over 4000 SNPs differentiated this *B. suis* bv. 5 cluster from the other *B. suis* biovars, while the difference to the *B. pinnipedialis* and *B. microti* reference strains merely amounted to 2607 and 2720 SNPs, respectively.

### Antimicrobial susceptibility testing

Strain 08RB3647 was tested for susceptibility to antibiotics of different classes: aminoglycosides (gentamicin, streptomycin), cephalosporins (ceftriaxone), fluoroquinolones (ciprofloxacin, levofloxacin), sulphonamides (sulfamethoxazole) and tetracyclines (doxycycline, tetracycline), as well as the antimicrobial compounds chloramphenicol and rifampicin. The MIC values for all substances were in the lower range of the tested concentrations, not exceeding 1 µg/ml, which was the highest tolerated concentration observed for chloramphenicol and rifampicin. When compared to the epidemiological cut-off values given by EUCAST, the strain could be classified as susceptible to all tested compounds (Table 5).

**Table 5:** Results of antimicrobial susceptibility testing of strain 08RB3647 including the tested concentration range of the antibiotics and the minimum inhibitory concentration (MIC). The resistance is evaluated according to EUCAST epidemiological cut-off values (ECOFFs) (S – susceptible).

Antibiotic	Tested range [µg/ml]	MIC [µg/ml]	EUCAST classification
Ceftriaxone	0.004–0.12	0.008	S
Chloramphenicol	0.5–16	1	S
Ciprofloxacin	0.002–4	0.25	S
Doxycycline	0.004–8	0.125	S
Gentamincin	0.004–8	0.125	S
Levofloxacin	0.004–4	0.25	S
Rifampicin	0.125–8	1	S
Streptomycin	0.008–16	0.5	S
Tetracycline	0.004–8	0.25	S
Trimethoprim/sulfamethoxazole	0.002/0.04–4/76	0.125/2.375	S

## Discussion

Human brucellosis cases are rare in the European Union, and in particular in Germany, where the mean annual notification rate was as low as 0.38/1,000,000 population between 2006 and 2018^5^. Also, it can be expected that *B. melitensis* is the most common agent of human brucellosis in Germany and Middle Europe, as this was the most frequently identified species in investigative studies^38,57^. However, in the majority of brucellosis cases, the species was not determined^5,15^, thus, the true prevalence of *Brucella* species remains elusive. For epidemiological investigations it would be desirable to not only identify the causative agent on the genus level, but also to mandatorily identify the species and possibly the biovar. However, this would require close collaboration between clinics, contract laboratories, authorities and the reference laboratories, as not every laboratory has the capacity for in-depth investigation of isolates.

For the human brucellosis case presented in this study *B. suis* was identified as infecting agent. This species comprises five biovars, that differ in host specificities and prevalence in human brucellosis cases. In the period between 2015 and 2019 only a single human case of *B. suis* infection has been reported to the authorities^15^. The latter was a patient from Germany who was infected by *B. suis* bv. 1^7^. This biovar is more common in Latin America^58^. In Europe, several human infections with *B. suis* bv. 2 have been reported. This biovar is endemic in wild boars, pigs and hares^18–20,59^, why hunters and farmers are predominantly affected^6,60^. *B. suis* bv. 3 infections are reported from patients in China^4^. Most human *B. suis* bv. 4 infections were found in northern Canada^22^. Whatmore et al.^61^ also list a human isolate from 1979 originating from Finland.

Little information is available on isolates of *B. suis* bv. 5. Since the 1960s, atypical *Brucella* strains have been isolated from mouse-like rodents (Murinae) in the northern foothills of the Greater Caucasus, which, at that time, could not be unambiguously positioned within the known *Brucella* taxonomy^23,62^. These isolates differed from other *B. suis* biovars regarding their growth characteristics, as they are fast-growing like the "non-classical" *Brucella microti*, whose primary hosts are also rodents^63^, and reactions in biochemical tests^64^, but their pathogenicity and differential characteristics are matching those of *B. suis* isolates^23,62,65^. Therefore, the Subcommittee on Taxonomy of *Brucella* of the International Committee on Systematic Bacteriology postulated a new *B. suis* biotype for these strains, biovar 5, in 1982^66^. A couple of years later, the reference strain *B. suis* bv. 5 513, which was isolated from a house mouse (*Mus musculus*)^24^, was deposited under the number NCTC 11996 in the National Collection of Type Cultures, Colindale, London^67^, despite the fact that Vershilova et al.^62^ proposed a different strain, 514, as type strain. The reference strain 513 is one of two strains which is commonly used in the literature when in need of a *B. suis* bv. 5 strain. The other being ELT80 (sometimes also called BCCN#R29 or BCCN 82.75). The origin of this strain cannot be determined for certain, as some studies state that the strain was isolated from a patient in England^34,68^, while others place the geographic origin in the USA^31–33^ or state that it is unknown^49^. Due to this small number of available bv. 5 isolates, one of these two strains, 513 or ELT80, is usually used for the development of *Brucella* sp. detection assays, e.g. Bruce-ladder PCR or Suis-ladder^31,33^, or genotyping approaches, e.g. MLVA^49^. There are no comprehensive studies applying these assays on several isolates of this biovar. Isolate 08RB3647 showed a deletion in one of the loci that is targeted in the Suis-ladder PCR. This prevented the unambiguous identification of the strain by PCR. Since no other *B. suis* bv. 5 field strains were available, it cannot be assessed whether this deletion is common among the strains of this biovar. However, deletion or modifications in genomic loci which are targeted by diagnostic PCRs are known from other pathogens, e.g. *Burkholderia mallei*^69^, and investigators should be aware of the possibility of false conclusions based on these assays.

Therefore, in the present study, the identity of the human isolate 08RB3647 as *B. suis* bv. 5 strain could only be unambiguously determined by genome sequencing and subsequent genotyping. Although the isolate was also correctly identified using classical phenotyping, this method is reserved for specialized laboratories due to the limited availability of the required monospecific sera.

The species *B. suis* belongs to core clade of *Brucella* phylogeny^70^ and in contrast to other *Brucella* species, e.g. *B. melitensis*^37,71^, strains of the same biovars form distinct clusters in the polytomy. From the beginnings of *Brucella* genotyping, allele-based typing methods already indicated that *B. suis* bv. 5 is quite distinct from the other *B. suis* biovars^49,72^. This was confirmed by whole genome comparison, in which *B. suis* bv. 5 displayed higher concordances with *B. microti* CCM4915 and *B. pinnipedialis* strains, while other *B. suis* biovars formed one cluster at a different branch of the phylogenetic tree^70^. This higher genomic similarity to *B. microti* might have led some authors to deeming *B. suis* bv. 5 as “misidentified” species^73^. However, this similarity was also found in the present study for strain 08RB3647 and other *B. suis* bv. 5 strains based on SNP typing. Due to the lack of sequencing data of field isolates, only the reference strains 513 and 513 UK and one other *B. suis* bv. 5 isolate, CVI_73, could be used for comparison. There is little information on the origin of the latter strain. Duvnjak et al.^20^ state that this strain was collected in 2004 in Slovenia from an animal. Regarding the 28 SNP difference between this strain and the reference strains, it can be assumed that CVI_73 is not the strain 513, which was passaged through an animal. Interestingly, the human isolate 08RB3647 showed a higher average nucleotide identity to the Slovenian isolate, while in SNP typing the similarity was higher to the reference strains. This might result from misassemblies in the genome. Nevertheless, the differences in SNP typing exceed by far the threshold of 5 to 7 SNPs, which is often applied for the definition of closely related strains^74,75^. It can be concluded, that strain 08RB3647 is most likely a field isolate and not the reference strain *B. suis* bv. 5 513.

There is a lack of knowledge on the pathogenicity of *B. suis* bv. 5 to humans. To our knowledge, the only report on a human brucellosis outbreak caused by *B. suis* bv. 5 was published by Repina et al.^76^ in 1993. The authors report that a family and two neighbours were diagnosed with brucellosis after contact with the domestic cat owned by the family. *Brucella* isolates were obtained from one patient and the cat and differential characterization identified *B. suis* bv. 5. The authors hypothesized that the urine of the cat promoted the spread of *Brucella*. The exact location was not mentioned in the report, but with regard to the involved institutions from e.g. Novosibirsk, it can be expected to be in South West Siberia.

To the authors’ knowledge, this is the first study reporting MICs of *B. suis* bv. 5 for antimicrobial substances determined by microdilution method. The susceptibility profile observed for strain 08RB3647 was comparable to the MICs found for *B. melitensis* strains as given by EUCAST. However, comprehensive data on antimicrobial susceptibility of *B. suis* is lacking and should be addressed in future studies.

The source of the human infection reported in the present study remains elusive. It can be assumed that the infection was contracted by close contact to an infected animal. A more thorough determination of the geographic origin of strain 08RB3647 is hampered by the lack of available sequence data of this biovar. Also, to our knowledge, there are no studies on the geographic distribution of *B. suis* bv. 5. However, as in case of the species, the biovars of isolates are not always determined and the prevalence of biovars might be underestimated. Due to this gap, we cannot state whether the deletion observed in one of the Suis-ladder target loci is of general importance for the PCR-based identification of this biovar. Nevertheless, microbiologists should be aware of the problems caused by mutations in the pathogen’s genome that could prohibit correct diagnosis by PCR.

### Supplementary Information

Supplementary Information.

## Data Availability

The datasets generated and analysed during the current study are available in the European Nucleotide Archive repository, under the BioProject number PRJEB62596.
